# Identification of *Rana dybowskii Ferritin-Heavy chain* gene and analysis of its role during bacterial infection

**DOI:** 10.1371/journal.pone.0337205

**Published:** 2025-12-16

**Authors:** Huimin Ren, Ye Liu, Yutong Liu, Yiming Liu, Hina Hassan, Yufen Liu, Peng Liu, Wenge Zhao

**Affiliations:** College of Life Science and Technology, Harbin Normal University, Harbin, China; Versiti Blood Research Institute, UNITED STATES OF AMERICA

## Abstract

Ferritin is widely present in organisms, which can maintain iron relatively stable and participate in the immune response. In this study, the full-length coding sequence (CDS) of the *Rana dybowskii* (*R. dybowskii*) *Ferritin-Heavy Chain* (*Fer-H*) gene was cloned by the polymerase chain reaction (PCR) method and characterized by bioinformatics analysis. In order to further explore its role, the inflammation model was established by using *Aeromonas hydrophila* (*Ah*). The activity of antioxidant enzymes in some tissues was detected, and the expression level of the *R. dybowskii Fer-H* (*RdFer-H*) gene was detected by quantitative real-time PCR and Western blot analysis. Bioinformatics analysis revealed that the *Fer-H* gene was 534 bp long, encoding 177 amino acids, and there was a Pfam Ferritin domain. When compared to other species with the same nucleotide sequence, *Rana temporaria* has the highest homology (94%) with the *Fer-H* gene. The activities of antioxidant enzymes indicated that the activities of SOD and CAT increased significantly, while the activity of GSH-Px decreased distinctly. This meant that the bacterial infection had caused serious oxidative damage to *R. dybowskii*. The qRT-PCR results confirmed the broad expression of the *Fer-H* gene in all *R. dybowskii* tissues. Furthermore, the transcription level was significantly up-regulated after bacterial infection, and the protein accumulations were consistent with the transcript levels in liver and muscle tissue according to Western blot after *Ah* infection. This study hypothesizes that the *Fer-H* gene contributes to *R. dybowskii*’s immune response during bacterial infection. It also broadens the research idea for exploring the anti-infection immune response mechanism of amphibians.

## Introduction

In 1937, Laufberger purified *Ferritin* (*Fer*) from a bull’s spleen by crystallizing with cadmium salts, and this was the first description [1]. When iron is deficient, Fer can bind to excess free Fe^2+^ in the cellular environment and release it, effectively controlling intracellular iron and preventing cell damage [2]. Research had demonstrated that ferritin was upregulated during bacterial and viral infections in fish, as an acute-phase protein, playing a critical role in immune protection [3,4]. Additionally, the H and L subunits of ferritin act synergistically to facilitate the entry of Fe²⁺ into ferritin, where it is oxidized to Fe³⁺ for storage to contribute significantly to antioxidation [5]. Except for vertebrates, ferritin is also involved in immune regulation in certain invertebrates and plants. Pacific white shrimp injected with recombinant ferritin exhibit enhanced antiviral capabilities [6]. Similarly, under cold or drought, plants modulate ferritin expression to mitigate disturbances, thereby improving stress tolerance and preventing damage [7]. These findings provide reference basis for the pathological mechanisms of ferritin in inflammatory diseases.

*R. dybowskii* belongs to the *Ranidae Rana*, and is a dominant species with a strong ability to resist cold environments in Northeast China. The fallopian tubes of adult female *R. dybowskii* can be used to prepare Oviductus ranae, a kind of traditional Chinese medicine. It is also useful for food because it is rich in a variety of substances with high nutritional value [8]. In recent years, with the continuous expansion of the forest frog breeding industry, higher breeding density often leads to the occurrence of some infectious bacterial diseases. *Ah* infection, among others, causes “red leg syndrome”. It is an important reason for the substantial reduction of the amphibian population [9]. In light of this situation, the study on the *R. dybowskii Fer-H* gene may offer suggestions for disease prevention in breeding.

In this study, we cloned *Fer-H* cDNA to obtain the coding sequence of *R. dybowskii* and then established the *R. dybowskii* inflammation model. Meanwhile, we observed the pathological tissue characteristics. Subsequently, we detected the antioxidant enzyme activity during inflammation. Finally, we analyzed the transcriptional and translational levels of the *Fer-H* gene after infection by qRT-PCR and Western blot technology. The study provides a foundation for investigating the immune function of the *Fer-H* gene and offers a new theoretical basis for disease prevention, artificial breeding, and the protection of *R. dybowskii*.

## Materials and methods

### Animals and bacterial strain

The animal experiment involving *R. dybowskii* was approved by the laboratory animal care Committee of Harbin Normal University (HNUARIA2021002) and performed in accordance with the Regulations for the Administration of Affairs Concerning Experimental Animals. *R. dybowskii* were raised and tested according to ethical standards.

The healthy *R. dybowskii*, weighing 16 ± 2 g, were captured from the Artificial Breeding Farm in A-cheng District, Harbin, Heilongjiang Province, China, and subsequently reared in the Laboratory of Biochemistry and Molecular Biology at Harbin Normal University. After fasting for a week, we performed the corresponding operations according to the requirements of experimental ethics. The *Ah* strain dw1701–1909 was isolated and identified by our laboratory [10].

### Cloning and bioinformatics analysis of *Fer-H* gene

We used the *Fer-H* sequence of the *Rana temporaria* as a reference (GenBank: XM_040328412) to make primers using Primer Premier 5.0 software after aligning the nucleotide sequence. The primer sequences used for PCR amplification are listed in Table 1. The PCR reaction was performed using cDNA synthesized from *R. dybowskii* liver tissue as the template. The amplified products were purified using a gel extraction kit and subsequently ligated into the pMD18-T vector (Takara Bio, Dalian, China) following standard protocols. The coding region of the *RdFer-H* gene (GenBank: ON815293) was deposited in 2022 as part of a preliminary study.

**Table 1:** Primer information used in the experiment.

Primer name	sequence (5′ → 3′)	Length
Fer-H-F	AACAGTGATTGGACGGAACC	534 bp
Fer-H-R	AGAGGATCTCAGTCGTGGGA	
Fer-H-q-F	TTCCTCCACGCTGGTTCT	172 bp
Fer-H-q-R	CAATCGGCAGGTCAATCT	
β-actin-F	AAGAATGAGGGCTGGAACA	172 bp
β-actin-R	GTGCGTGACATCAAGGAGAAGC	

The recombinant plasmids were transformed into competent *Escherichia coli* DH5α cells. Positive clones were screened by colony PCR, and verified clones were sequenced by Shanghai Sangon Biotech Co., Ltd.

The obtained *Fer-H* coding sequences were analyzed using DNAMAN 6.0 (Lynnon Biosoft, USA) and MEGA7 for sequence alignment and phylogenetic analysis. Reference species used for comparison are listed in Table 2. Additional methodological details can be found in Wu et al. [10].

**Table 2:** Reference species and sequence information.

Species	Accession No. of nucleotide	Accession No. of amino acid
*Rana temporaria*	XM_040328412	XP_040184346.1
*Bufo gargarizans*	DQ437112.1	ABD75379.1
*Nanorana parkeri*	XM_018565189	XP_018420691.1
*Rana catesbeiana*	BT081921.1	ACO52052.1
*Xenopus laevis*	AF538970	AAQ10928.1
*Xenopus tropicalis*	NM_203677.1	NP_989008.1
*Alligator sinensis*	XM_006019073.1	XP_006019135
*Larimichthys crocea*	FJ788423.1	ACY75475.1
*Danio rerio*	AF295373_1	AAG37837.1
*Salmo salar*	NM_001123657	NP_001117129.1
*Oncorhynchus mykiss*	NM_001124547	NP_001118019.1
*Psetta maxima*	GU182880	ADI24353.1
*Ictalurus punctatus*	NM_001200338.1	NP_001187267.1
*Oreochromis niloticus*	XM_003445695.4	XP_003445743.1
*Gallus gallus*	NM_205086.2	NP_990417.1
*Homo sapiens*	AF088851	AAF89523.1
*Mus musculus*	X52561.1	CAA36795.1
*Oryctolagus cuniculus*	XM_008274404	XP_008272626.1
*Ovis aries*	NM_001009786	NP_001009786.2
*Bos taurus*	NM_174062.4	NP_776487.1
*Sus scrofa*	NM_213975.1	NP_999140.1
*Andrias davidianus*	JX195179.1	AFQ59980.1

### Inflammation models

Before the experiment, *R. dybowskii* should be acclimated at a room temperature of 20 ± 2°C (adjusted with air conditioning) for one week. Animals should be inspected, cleaned, and provided with fresh water, with crickets offered daily as feed. Care should be taken to avoid causing pain due to unnecessary handling.

*Ah* (DW1701–1909) was cultured in the bacterial solution to a concentration range of 0.4–0.6 detected by OD600. Based on the median lethal dose (LD50) found in lab studies [11], we diluted the bacterial suspension to 1.5 × 10^7^ CFU/mL and kept it at 4°C until we needed it again. We randomly divided *R. dybowskii* into two groups: an experimental group and a control group, with 30 frogs in each group. Every frog in the experimental group (*Ah*) was injected intraperitoneally with 1 mL bacterial suspension (1.5 × 10^7^ CFU/mL), the control group was injected with 1 mL LB liquid culture medium (1% NaCl, 0.5% yeast extract, 1% peptone, 100 mL ddH_2_O).

*Ah* produces a highly virulent exotoxin and is the main pathogen responsible for amphibian Red-leg disease which will come on rapidly, with near-death frogs potentially appearing before scheduled sampling time points. To minimize animal suffering, experimenters examined the frogs three times daily for clinical signs of Red-leg disease, including lethargy, bloody mucus at the mouth or anus, redness and swelling at the tips of the hind toes, and bright or dark red hemorrhagic spots on the hind limbs [12]. Upon detecting these symptoms, euthanasia was promptly administered to alleviate the R. dybowskiis’ suffering.

Benzocaine is commonly used to euthanize fish and amphibians [13–15]. Based on a reference concentration of 200–300 mg/L [15], a concentration of 200 mg/L benzocaine was used to anesthetize *R. dybowskii*, which weighed approximately 16 ± 2 g. Benzocaine was dissolved in a small amount of ethanol before use, and the pH of benzocaine was adjusted to 7.0 with 1 M NaHCO_3_. The *R. dybowskii* frogs were placed in the benzocaine for 20 minutes. During this period, eye movements, knee reflexes, and heartbeat and respiration were observed to see if they stopped, and The *R. dybowskii* frogs were considered to be euthanized if they did not respond to the above reactions. Individual tissues were collected at different times after ensuring euthanasia of the *R. dybowskii*.

Samples of heart, liver, spleen, lung, kidney, stomach, skin, and muscle tissue of *R. dybowskii* were collected at 6 h, 24 h, 48 h, 72 h, and 120 h, respectively, after injection. Three samples were collected at each time point. The tissue samples were fixed for 24 h in 10% formalin fixative for histological analysis.

### Histology

The liver, muscle, and skin samples were dehydrated in gradient ethanol and xylene, and then embedded in paraffin. Serial sections (5 μm) were mounted on slides coated with protein glycerol. Sections were stained with hematoxylin and eosin (HE) for general histologic examination.

### Determination of antioxidant enzyme activity

The liver, spleen, kidney, and muscle tissues of *Ah* and the control group were determined at 6 h, 24 h, 48 h, 72 h, and 120 h after infection. The total protein concentration (TP) (Nanjing, Jiancheng, China, A045-2–1), superoxide dismutase (SOD) (Nanjing Jiancheng, China, A001-3–1), catalase (CAT) (Nanjing Jiancheng, China, A007-1–1), and Glutathione peroxidase (GSH-Px) (Nanjing Jiancheng, China, A005-1) activities of the samples were determined according to the kit instructions and repeated three times. EXCEL 2019 calculated the antioxidant enzyme activity data according to the kit instructions.

### Quantitative Real-time PCR detection

After 6 h, 24 h, 48 h, 72 h, and 120 h of infection, the cDNA of the heart, liver, spleen, lung, kidney, stomach, skin, and muscle of *R. dybowskii* were selected from the control and *Ah* groups as the qRT-PCR template. Fer-H-q was used as a primer (Table 1), and the β-actin gene was used as the internal reference gene for qRT-PCR. The reaction procedure was performed according to the ChamQ Universal SYBR qPCR Master Mix instructions (Vazyme, Nanjing, China) and set up experimental replication three times. The results were calculated using EXCEL 2019 with the 2^–ΔΔCT^ method.

### Western blot detection

The following antibodies were used in this study: Rabbit polyclonal anti-Ferritin Heavy Chain (1:1,000 dilution; catalog #DF6278; Affinity Biosciences, Beijing, China), Rabbit polyclonal anti-β-actin (1:2,000 dilution; catalog #AF5003; Beyotime Biotechnology, Shanghai, China), HRP-conjugated AffiniPure Goat Anti-Rabbit IgG (H&L) (1:5,000; catalog #bs-40295G-HRP; Bioss, Beijing, China).

The total protein was extracted using a RIPA lysis buffer mixture (RIPA : PMSF = 100 : 1) and quantified the product by BCA method. After SDS-PAGE electrophoresis, the products were transferred to a PVDF membrane and blocked with 5% skim milk powder at room temperature for 1 hour. Next, we incubated the products with the primary antibody overnight at 4°C and rinsed them with TBST three times, then incubated them again with the second antibody at room temperature for 2 h and washed them with TBST three times again. Finally, we exposed and developed the product using enhanced chemiluminescence (ECL), and then analyzed the gray values of the strips using Image J software.

### Data analysis

All the above data were presented as means ± standard error of the mean (SEM). Three frogs were provided for each mean value. A one-way analysis of variance was used to test the difference between the experimental group and the control group. The two-way ANOVA was used for the comparison between the groups. Statistical analysis for SPSS 26.0 (IBM, USA) was used to compare the data with a significant difference. Differences were considered statistically significant if P < 0.05.

## Results

### Cloning of the *RdFer-H* gene

Total RNA from the liver tissue of *R. dybowskii* was extracted by the Trizol method and then detected by 1.0% agarose gels, with clear bands of 28S and 18S. The OD260/OD280 values of the total RNA detected by the UV spectrophotometer were in the range of 1.8–2.0, which were in accordance with the standard and proved RNA integrity. The *Fer-H* gene was amplified after transcription, and the target fragment was about 530 bp after 1.0% agarose gels. The objective gene was ligated into the pMD18-T vector and transformed into E. coli DH5α, which was up to standards after colony PCR and sequencing.

### Bioinformatic analysis of the *RdFer-H* gene

**Fig 1:**
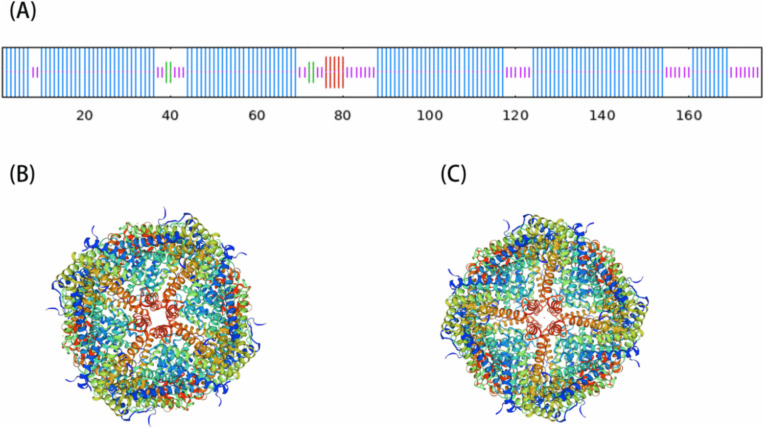
RdFer-H structure. **(A)** Prediction of the secondary structure of the RdFer-H amino acid sequences by SOPMA online software. The blue lines, the red lines, the purple lines, and the green lines represent α-helices, extended strands, random coils, and β-turns. **(B)** A tertiary structural model of RdFer-H from *R. dybowskii*, constructed using the online modeling tool SWISS-MODEL. **(C)** Three-dimensional structural model of mouse ferritin (3wnw.1. A).

The nucleotide sequence of the *RdFer-H* gene was obtained by sequencing (NCBI accession number: ON815293). The coding region of the *RdFer-H* gene is 534 bp in length and encodes 177 amino acids. The SOPMA online software predicted that the RdFer-H mostly had an α-helix (74.01%), an irregularly coiled (20.90%), an β-turned (2.82%), and an extended strand (2.26%) structure (Fig 1A). The online modeling tool SWISS-MODEL was used to construct the three-dimensional (3D) structural models of the RdFer-H. It predicted a strong consistency in tertiary structure between the mouse Fer-H protein and the RdFer-H protein (Fig 1B), and it found that the amino acid sequences of the RdFer-H protein shared an 86.53% sequence identity with the mouse Fer-H protein; the GMQE was 0.92. It’s clear from the three-dimensional structure that the RdFer-H protein is mostly made up of α-helices, irregularly coiled and extended chains, and a hollow, globular protein, which is mostly in line with the secondary structure.

**Fig 2:**
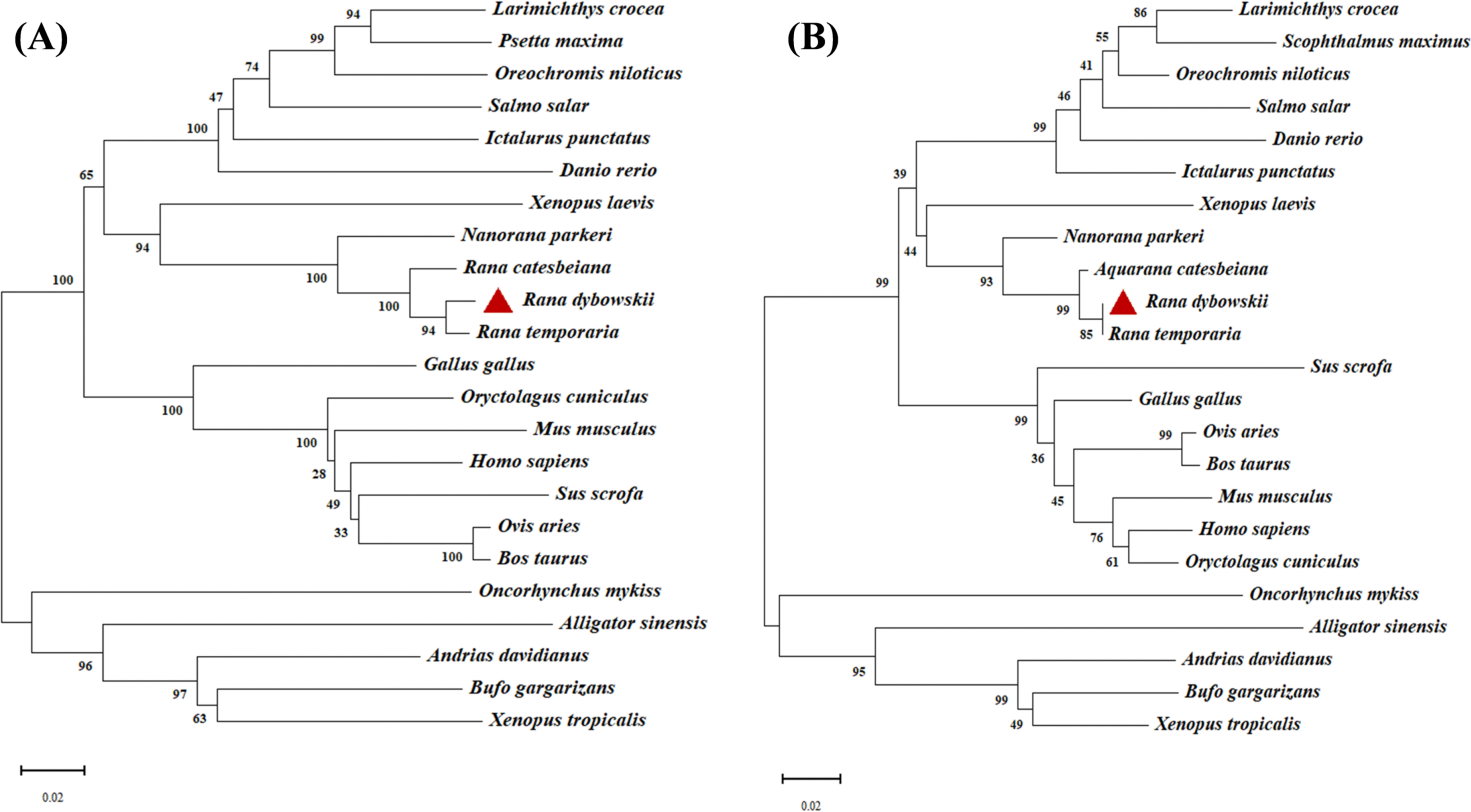
Phylogenetic analysis of RdFer-H and other Fer-Hs from animals constructed by MEGA (version 11). The scale bar (number of substitutions/site) corresponds to the relative branch length. *Fer-H* of *Rana dybowskii* was marked with a triangle. (**A**) nucleotide phylogenetic tree, (**B**) amino acid phylogenetic tree.

To explore the relationships of the *Fer-H* gene between *R. dybowskii* and various organisms, the phylogenetic tree of RdFer-H at the nucleotide level was constructed (Fig 2A). The topology tree featured two major clades, which further divided into five subclusters: fish, amphibia, reptiles, aves, and mammalia. RdFer-H protein was most closely related to *Rana* *temporaria* (97%) among amphibians, and the homology with fish (74% ~ 92%) was relatively high, while the homology with mammals (44% ~ 65%) varied greatly but was still relatively stable.

Using MEGA 7.0 software, another phylogenetic tree at the amino acid level was also constructed between *R. dybowskii* and the reference species (Fig 2B), which indicated that *R. dybowskii* is in the same smallest branch as *Rana temporaria* and is most closely related, followed by *Rana catesbeiana*. The three species enter a branch, and then they form a close relationship with Nanorana parkeri, but they are relatively distant from the other species.

### Pathologic changes in *R. dybowskii*

**Fig 3:**
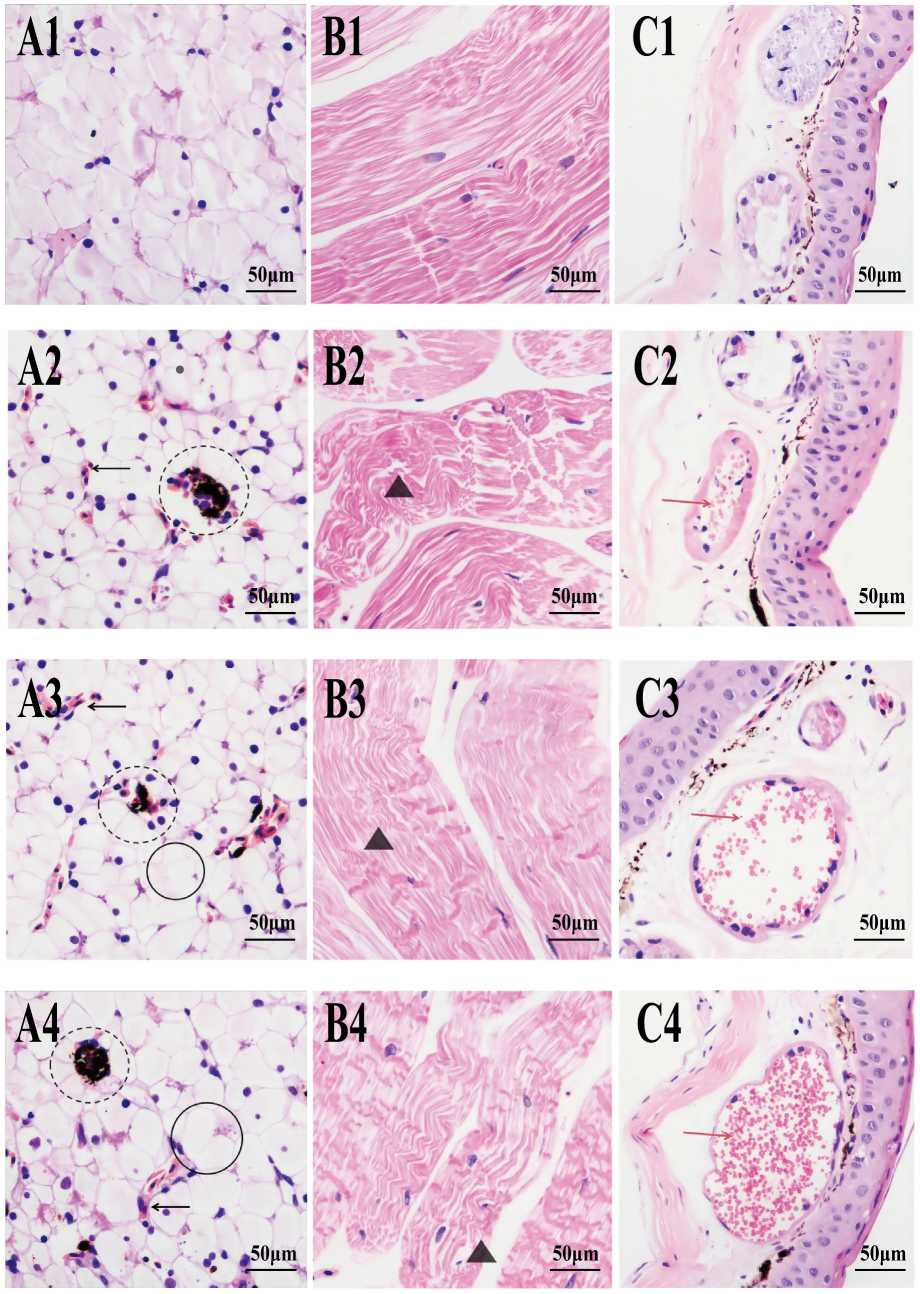
Tissue sections of *Ah*-treated *R. dybowskii.* Scale bars represent 50 μm. Slides were viewed under a light microscope at 400 × magnification. (A: Liver B: Muscle C: Skin 1: Control 2: 6 h 3: 24 h 4: 48 h) In A, Dotted circles indicates a melanin macrophage, the black arrow indicates a hepatic blood sinusoid, and the solid circle indicates cellular vacuolization. In B, the black triangle indicates the disintegration of a muscle fracture. In C, the red arrow indicates a red blood cell in the lumen of the glandular follicles.

Pathological changes of the *R. dybowskii* liver, muscle, and skin were observed after infection at 6 h, 24 h, and 48 h (Fig 3). Vacuolated cellular lesions, blood sinusoids, and melanin macrophages were found in the liver tissue of the 6 h experimental group (Fig 3. A2). As the infection time prolonged (24 ~ 48 h), the vacuolization of liver tissue was gradually severe, and the distribution of blood sinusoids was gradually increased (Fig 3. A3 ~ A4). In muscle tissue (6 ~ 48 h), widening of the interstitial space and structural disorder, deformation, and disintegration of muscle fibers could be observed (Fig 3. B2 ~ B4). When *R. dybowskii*’s skin tissue was infected, the glandular follicle lumen was filled with a large number of erythrocytes and bluish-purple basophilic granulocytes with light pink collagen fibers (Fig 3. C2 ~ C4). This demonstrated the successful establishment of the *R. dybowskii* inflammation model.

### Changes of antioxidant enzyme activities in *R. dybowskii*

**Fig 4:**
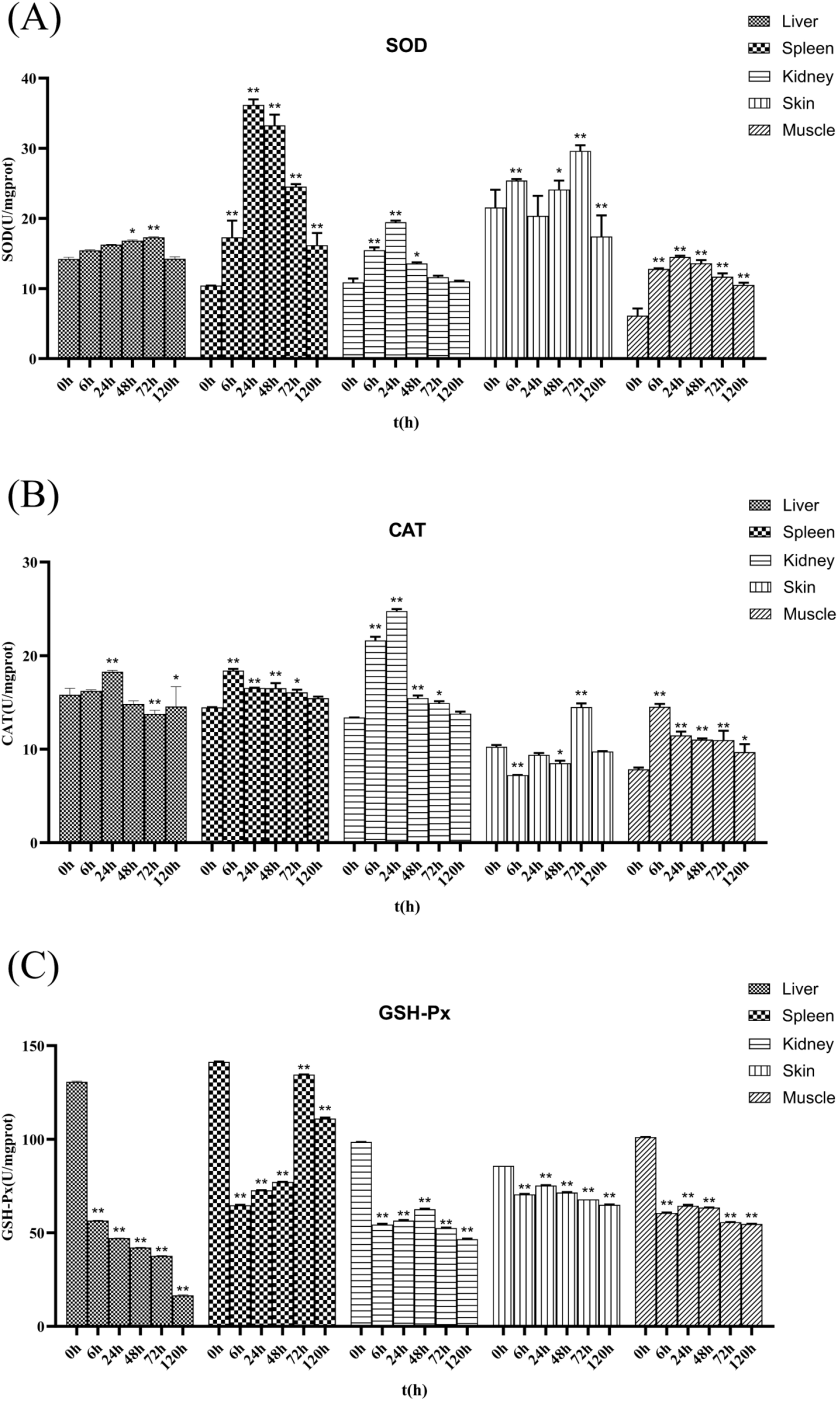
Changes of antioxidant enzyme activities in *R. dybowskii* after *Ah* treatment. The total protein content was determined at 595 nm using the TP method, and then the absorbance of the material was measured at different wavelengths according to the instructions of the kit, and the enzyme activity was calculated. *: Significant difference between experimental and control groups (P < 0.05); **: Highly significant difference between experimental and control groups (P < 0.01).(A) Changes in SOD activity in different tissues (B) Changes in CAT activity in different tissues (C) Changes in GSH-Px activity in different tissues.

After the *Ah* infection, antioxidant enzymes were detected in the liver, spleen, kidney, skin, and muscle tissues of *R. dybowskii*. As shown in Fig 4, the spleen, kidney, and muscle tissues had the highest SOD activity 24 h after infection, which was 2.47, 0.79, and 1.37 times higher than in the control group (P < 0.01). On the other hand, the liver and skin tissues didn’t have the highest SOD activity until 72 h after infection, when they were 0.19 and 0.37 times higher than in the control group (P < 0.01). At 6 h after infection, the CAT activity of the spleen and muscle tissues was 0.27 and 0.85 times higher than that of the control group (P < 0.01), and it kept going down as the infection time went on, but both were still significantly higher than those in the control group (P < 0.01). The CAT activity of the liver tissues was 0.16 times higher than that of the control group at 24 h after infection, and it went down for (48 ~ 72h) before going up a little at 120 h. At 72 h, the CAT activity in skin tissue reached its peak, which was 0.31 times that of the control group (P < 0.01). The CAT activity of kidney tissues showed a complete trend of first increasing and then decreasing, reaching the highest level 24 h after infection, which was 0.85 times higher than the control group (P < 0.01). The activity of GSH-Px in liver, kidney, skin, and muscle tissues all reached their lowest values after 120 h of infection, which were 0.13 (P < 0.01), 0.47 (P < 0.01), 0.76 (P < 0.01), and 0.54 (P < 0.01) times higher than those in the control group, respectively. In summary, the liver, spleen, kidneys, skin, and muscle tissues all demonstrated varying degrees of oxidative damage. The activities of SOD and CAT showed a trend of first increasing and then decreasing with the extension of infection time, but they were generally higher than those of the control group. Compared to the above two enzymes, GSH-Px activity had a decreasing trend and was lower than that of the control group.

### Transcription levels of the *RdFer-H* gene under physiological conditions

**Fig 5:**
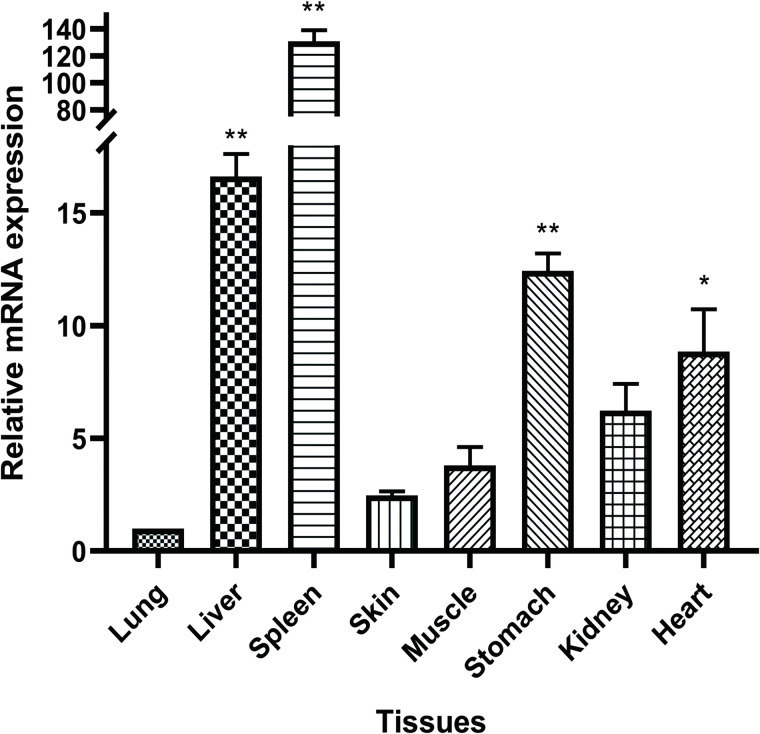
The gene expression profiles of *Fer-H* in different tissues of *R. dybowskii* under physiological conditions. The transcripts of *RdFer-H* in the lung, liver, spleen, skin, muscle, stomach, kidney, and heart were quantified by qRT-PCR. Values are presented as the mean ± standard deviation of three independent experiments. *P < 0.05, **P < 0.01. The relative expression in the lungs was used as an internal control.

The mRNA level of *RdFer-H* was analyzed in eight different tissues using qRT-PCR. As shown in Fig 5, the relative expression level of *RdFer-H* mRNA in lung tissue was significantly lower than that of other tissues (lung expression as a control). The expression of *RdFer-H* is highest in the spleen (P < 0.01), followed by the liver (P < 0.01), stomach (P < 0.01), heart (P < 0.05), kidney, muscle, and skin tissues, exhibiting tissue-specific changes.

### Transcription levels of the *RdFer-H* gene under *Ah* infection

**Fig 6:**
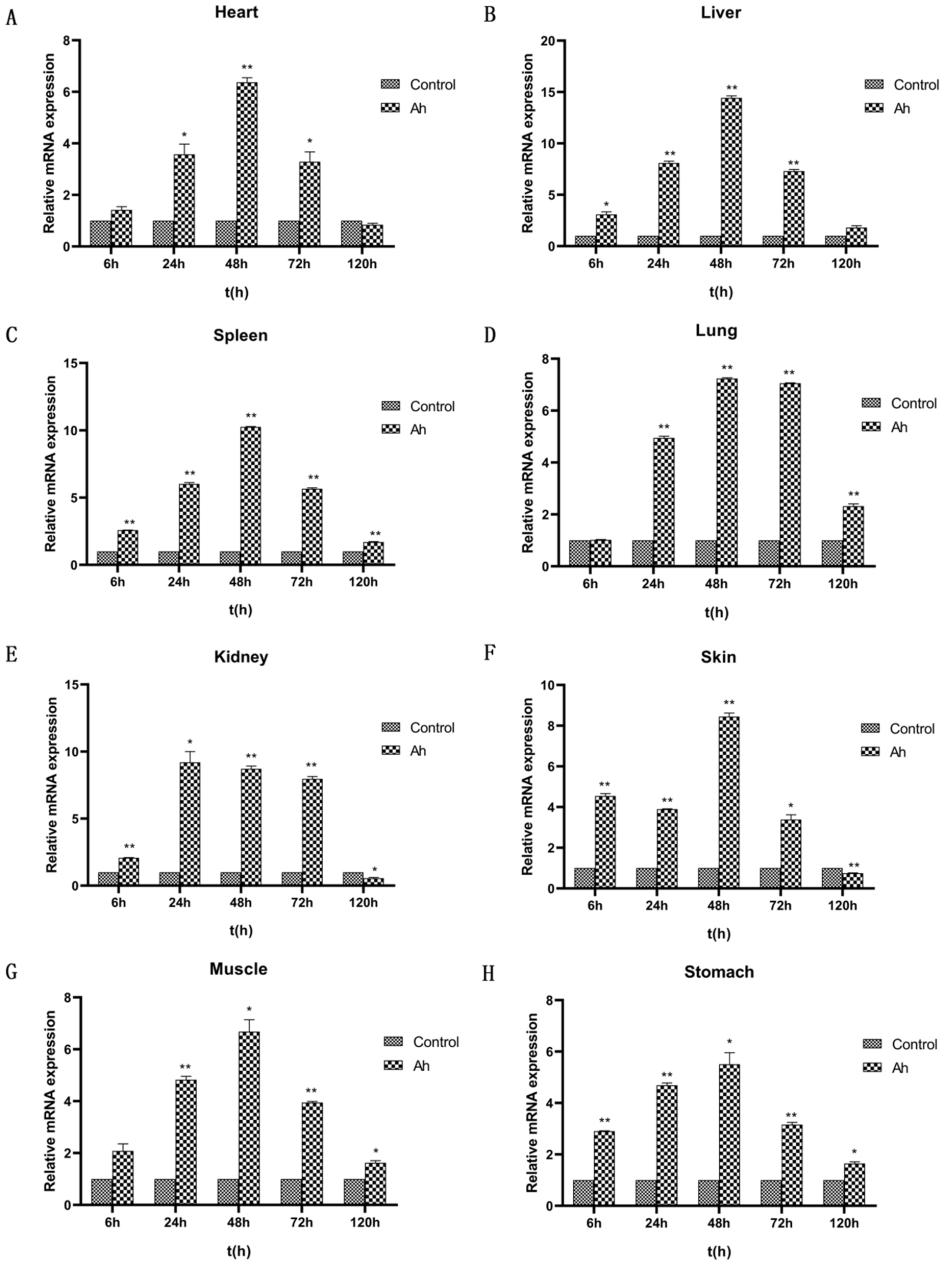
Expression changes of the *Fer-H* gene in *R. dybowskii* under *Ah* infection. After injection of *Ah* in *R. dybowskii*, samples of eight tissues were collected at five time points: 6 h, 24 h, 48 h, 72 h, and 120 h, and gene expression changes were detected using qRT-PCR. The control group was injected with LB liquid medium; β-actin was used as an internal reference. *: Significant difference between experimental and control groups (P < 0.05); **: Highly significant difference between experimental and control groups (P < 0.01).

As shown in Fig 6, in the *Ah* experimental group, the transcription levels of the *RdFer-H* gene in various tissues displayed different patterns. In heart tissue, the expression of the *Fer-H* gene initially increased before decreasing. The expression peak was observed at 48 h post-infection, reaching 6.37 times higher than that of the control group (P < 0.01). Subsequently, the expression declined and reached 0.84 times that of the control group at 120 h after infection (P < 0.01). Similarly, the *Fer-H* genes in liver, stomach, spleen, and muscle tissues exhibited a similar trend to that in heart tissues, reaching peak expression levels at 48 h post-infection. These levels were 14.43, 5.50, 10.25, and 6.68 times higher than the control group, respectively. The expression levels remained elevated compared to the control group during the entire duration of the infection (72–120 h).

In lung tissues, *Fer-H* gene expression was similar to the control group at 6 h and peaked at 48 h (7.24 times higher than the control group, P < 0.01). The kidney tissues first responded to infection and reached peak expression at 24 h (9.20-fold higher than the control group). However, at 120 h, the gene expression level was lower than the control group (0.56 times, P < 0.01). After 6 h of infection, the skin tissue showed a significant increase in *Fer-H* gene expression, which increased by 4.54-fold compared to the control group. However, the gene expression showed a decreasing trend after 24 h, 3.89-fold higher than the control. At 48 h, this number increased sharply, 8.45-fold higher than the control. Subsequently, at 120 h, the expression level was down regulated, only 0.75-fold higher than the control group (P < 0.01). It indicated that different tissues have different response times and levels of the *RdFer-H* gene in response to inflammation.

### Detection of RdFer-H protein levels under *Ah* infection

**Fig 7:**
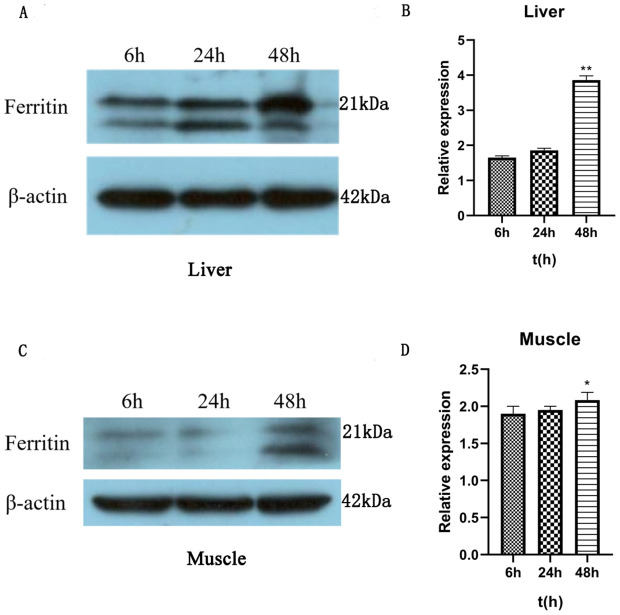
Ferritin expression after *Ah*infection. (A) and (C) are immunoblot analyses of liver and muscle cell extracts with the indicated antibodies. (B) and (C) are plots of changes in relative protein expression produced by gray value analysis using the software.

Western blotting in the experimental group detected RdFer-H protein in the liver and muscle tissues, revealing some changes (Figs. 7A, 7B, 7C, and 7D). The expression level of Fer-H protein in liver tissue was continuously upregulated from 6 to 48 h, with the highest amount at 48 h, which was 2.37 times higher than that at 6 h of infestion (P < 0.01). In muscle tissue, it was similar to that of liver tissue and exhibited the highest expression of Fer-H protein at 48 h. The results showed that the expression of RdFer-H protein was 1.14 times higher at 48 h than at 6 h (P < 0.05). The above results were basically consistent with the qRT-PCR analysis, suggesting that RdFer-H may be involved in the immune response induced by bacterial infection.

## Discussion

Ferritin is not only a highly conserved iron storage protein but is also widely recognized as an acute-phase protein in innate immunity. The regulatory network governing ferritin’s function during inflammatory infections—particularly the cross-talk between iron chelation and immune metabolism—remains a frontier area of current research [16–19]. In vertebrates, mammalian ferritins predominantly comprise H/L subunits, whereas teleost fish (such as *Salmo salar* and *Cynoglossus semilaevis*) are frequently documented with H/M subunits [20,21]. These subunits regulate iron uptake and storage during infection through specialized roles in oxidation and mineralization. In fish models, the regulation of ferritin expression in response to pathogen stimulation and its in vitro phenotypes have been documented. For instance, rock bream Ferritin-M exhibits transcriptional upregulation following LPS and pathogen stimulation, with its recombinant protein demonstrating iron chelation and DNA-protective activity [22]; all three subunits (H/M/L) of seahorse ferritin respond to immune stimulation [23]; while disruption of the eukaryotic ferritin domain in tongue sole Ferritin-M resulted in the loss of iron-binding and antimicrobial capabilities [2 1]. Although preliminary insights exist into the immunoresponsive mechanisms of fish ferritins, the response patterns and functions of amphibian ferritins—during bacterial infections remain poorly characterized. This study employs the amphibian *R. dybowskii* as a model to preliminarily investigate the expression dynamics and tissue distribution characteristics of its *Fer-H* under *Ah* infection. The aim is to provide foundational clues for subsequent functional validation and mechanistic elucidation.

Ferritin is an almost spherical shell with an internal hollow cavity diameter of approximately 8 nm, capable of accommodating up to 4500 iron atoms [24,25]. Vertebrate ferritin is composed of 24 subunits of heavy chain and light chain [26]. Despite sharing similar secondary structures, the H and L subunits originate from distinct gene families and exhibit varying expression ratios across various tissues [27]. Previous studies had confirmed that in healthy individuals, the L subunit dominated in the human liver and spleen, while the H subunit was predominant in the heart [28]. However, inflammation, differentiation, and development dramatically induced the ratio of H and L subunits [26]. It was found that the homology between H and L subunit sequences was relatively low, especially in functional domains [29]. Some studies have demonstrated that the H subunit has an iron oxidase center, primarily chelating and oxidizing intracellular free Fe^2+^, whereas the L subunit is responsible for the hydrolysis of iron, mineralizing Fe^3+^ into the nucleus, and then storing it. The interaction between the H subunit and the L subunit is crucial in the regulation of intracellular iron [29]. Fish and amphibians contain a third subunit, known as the M subunit, in addition to the H and L subunits [30]. The M subunit has both iron oxidase centers and iron nucleation sites, which means it can do the work of both the H and L subunits on its own [31].

In this study, the *Fer-H* gene nucleotide homology between *R. dybowskii* and *Rana temporaria* was 94%, with only nine different bases. The amino acid sequences, on the other hand, stayed the same. This showed that the *Fer-H* gene was very similar in *R. dybowskii* and *Rana temporaria*. Researchers also discovered that the *Fer-H* gene’s coding region, measuring 534 bp in length and encoding 177 amino acids in Chinese giant salamanders [32] and Nanorana parkeri [33], aligns with that of *R. dybowskii*. It was suggested that the *Fer-H* gene is relatively conserved among amphibians and may have a similar biological function. The secondary and tertiary structural analysis of the RdFer-H protein revealed that the α-helix composition accounted for 74.01%, and the α-helix with high chemical bonding energy is located inside the protein, which plays an important role in stabilizing the protein structure [34].

Changes in cellular structure and cellular dysfunction are the foundation for disease occurrence and development. Histopathological research can directly and accurately reflect the pathological changes of tissues and cells [35]. Studies has revealed that the liver significantly contributes to the immune response of amphibians to bacterial infections, suggesting that liver function could serve as a novel biomarker for the health effects of bacterial infections [36]. They also observed that the liver, skin, and muscles suffered severe damage when Chinese giant salamander iridovirus (GSIV) infected giant salamanders, with these organs being the primary targets of damage [37]. After infection with *Ah*, the outline of liver cell of *Rana amurensis* disappeared, with the nuclear shrinkage, and iron-hematin deposition can be detected. The epidermis and dermis layers of the skin was separated, and mucous glands and granular glands filled with blood cells [38]. Extensive cellular vacuolization damage has affected the liver tissues, and the presence of melanin macrophages is evident. This is consistent with the demonstrated role of macrophages as a unique biomarker in antibacterial responses [39], suggesting that *R. dybowskii* liver tissues exhibit a certain degree of antibacterial response. Pathological observations of hosts infected with *Ah* have been documented in numerous aquatic animal models. In the pathological examination of *Ah* septicaemia in *Trionyx sinensis* [40], gastrointestinal changes including intestinal mucosal hyperaemia, increased mucus secretion, and epithelial detachment have been clearly recorded, confirming that this bacterium can induce acute inflammatory changes in the gut. Furthermore, pathological studies on infected Nile tilapia revealed significant haematocytic infiltration in the submucosal layer of the gastrointestinal tract and oedema in the lamina propria [41]. This study conducted haematoxylin and eosin (H&E) staining to evaluate histological damage in the liver, skin, and muscle tissues of *R. dybowskii.* Future research will explore the inclusion of gastrointestinal tissues to comprehensively assess the extent of A. hydrophila-induced damage to the digestive tract.

However, the interplay between iron metabolism and immunity extends far beyond this. Iron acts as a key catalyst in the Fenton reaction, leading to the rapid generation of highly toxic hydroxyl radicals [42,43]. Thus, while limiting iron availability to pathogens, the host must also guard against iron-induced oxidative damage to its own tissues [16,44].

Antioxidant enzyme activity is an important indicator of animal health, reflecting their resistance to external stress [45]. The antioxidant responses (AOS) produced by organisms can neutralize and eliminate harmful toxins in the body [46]. SOD is the first line of defense against oxidative stress, while CAT protects cells from the effects of hydrogen peroxide induced oxidative stress [47,48]. Up-regulation of SOD and CAT as a reaction to oxidative stress triggered by pathogens is one of the primary antioxidant defence mechanisms [49]. Research has shown [48] that the liver, spleen, and kidney tissues are the main tissues involved in the fish immune response, as well as the main sites for synthesizing immune defense molecules, which are responsible for the removal of pathogens or other foreign objects. In this study, there were significant increases in SOD and CAT activities in *R. dybowskii*’s liver, spleen, and kidney from 6 to 120 h during *Ah* infection. The SOD activity of goldfish also showed a similar trend under *Ah* infection [50]. Studies that examined CAT activity in the liver and kidney of catfish [51] and the spleen tissue of rohu [52] infected with bacteria observed the same trend. In this study, the spleen had higher SOD activity after *Ah* infection than other tissues. This shows that the spleen of *R. dybowskii* is an important tissue for dealing with bacterial infection and antioxidant stress. GSH-Px is an important antioxidant marker in vertebrates; it can break down H_2_O_2_ and help keep cell membranes structurally and functionally intact [48,53]. GSH-Px activity was also significantly reduced when African clawed frog embryos were exposed to toxic environments [54].

SOD is an effective antioxidant enzyme that converts toxic superoxide into less toxic H₂O₂, which is subsequently reduced to water by CAT [55]. The significant increase in SOD and CAT activities observed in this study not only confirms the enhancement of antioxidant capacity in *R. dybowskii* following *Ah* infection, but also provides compelling evidence for a deeper understanding of the role of antioxidant enzymes in the bacterial infection process. Lipopolysaccharide (LPS), a component of the cell wall of Gram-negative bacteria, induces cell inflammatory response and release inflammatory cytokines when it enters the lungs, thereby suppressing GSH levels [56].

In this study, after the establishment of the *Ah* inflammation model, various tissues of the *R. dybowskii* can be observed a significant reduction in GSH-Px activity in all tissues of *R. dybowskii*, with the liver tissue exhibiting a rapid decline. This aligns with the observed cell lysis and vacuolization of hepatocytes in pathological tissues after infection.

Many amphibians fight inflammation by increasing the levels of IL-1β, IL-6, TNF-α, and IFN-γ [57,58]. This can change how blood cells are phagocytosed and how plasma proteins are killed [59,60]. The above inflammatory factors also upregulated ferritin [61]. In addition, iron is a nutrient required for the growth of pathogens. After bacterial infection, organisms will inhibit the growth and reproduction of pathogens by upregulating the expression of ferritin to bind more iron ions in the cells. The H subunit of ferritin can chelate free Fe^2+^ in the organism and oxidize it [62,63].

This study found that the *R. dybowskii Fer-H* gene was significantly upregulated (P < 0.01) in all tissues from 6 h to 120 h after *Ah* infection. This was especially true in the liver, spleen, kidney, skin, and muscle tissues. With the increase in inflammatory factors, there is an increase in ferritin. The ferritin H subunit began to bind to free Fe^2+^ inside cells, which stopped pathogens from getting the iron they needed to grow and reproduce. Research has shown that Fer-H protein may be a candidate immune molecule involved in the acute response phase, and most inflammation-related stimuli seem to preferentially upregulate H-type subunits [64]. Western blot assays in this study demonstrated an upregulation of Fer-H protein in liver tissue after *Ah* infection. The result further confirms the claim that the liver is the main organ for iron metabolism and ferritin synthesis [31]. Like qRT-PCR, Fer-H protein reached its highest level within 48 h of infection. This shows that *R. dybowskii*’s *Fer-H* gene expression was the same at both the transcription and translation levels. The study on *Ah* infections in *Mylopharyngodon piceus* found that muscle Fer-H protein levels peaked after 48 h [4], which is the same time frame that we used in this experiment.Therefore, from the perspective of functional integration, the strong upregulation of Fer-H we observe may not only directly mediate “nutritional immunity” but also indirectly attenuate the Fenton reaction by reducing the labile intracellular iron pool, thereby alleviating the oxidative stress levels we have observed [18,65,66]. This hypothesis tightly links iron metabolism, oxidative stress, and innate immunity [19]. We can assume that *R. dybowskii* enhances the expression of Fer-H proteins in tissues during bacterial invasion as a defense mechanism, based on the aforementioned findings.

Existing research indicates that deferoxamine (DFO), deferiprone (DFP), and their derivatives not only effectively reduce levels of the labile iron pool (LIP) within the body, but also play significant roles in regulating immune cell function. For instance, DFO enhances antimicrobial defense by stabilizing hypoxia-inducible factor 1-alpha (HIF-1α) [67]. In murine infection models, DFO treatment suppresses the oxidative burst of neutrophils induced by Gram-negative bacteria and reduces cellular apoptosis, suggesting immunomodulatory potential [68]. Furthermore, deferoxamine has been found to reduce the release of inflammatory mediators including IL-6 and TNF-α, thereby mitigating tissue damage caused by systemic inflammatory response syndrome (SIRS) [69].

In summary, by correlating bacterial infection, oxidative stress markers, and Fer-H expression, this study tentatively outlines a hypothesis: during the early stages of *Ah* infection, the host rapidly upregulates Fer-H to execute ‘nutritional immunity’—limiting pathogen proliferation by restricting intracellular free iron—while simultaneously mitigating oxidative stress-induced tissue damage. Despite these advances, the study has certain limitations. To more precisely validate the proposed integrative functional model of ferritin H in coordinating immune and oxidative homeostasis, future studies will focus on directly manipulating iron metabolism pathways in both in vivo and in vitro models. This will be achieved through pharmacological interventions (iron chelators and Fer-H inhibitors) and genetic approaches, ultimately elucidating the precise immunoregulatory functions of Fer-H.

## Conclusion

In this experiment, the coding region of the *Fer-H* gene of *R. dybowskii* was successfully obtained; it is 534 bp long and encodes 177 amino acids. The *RdFer-H* gene has the closest genetic relationship with that of *Rana temporaria*, followed by other amphibians and fish. Its genetic relationship with mammals is more distant, but it is highly conserved among different species. The liver and muscle tissues of *R. dybowskii* showed the highest expression of Fer-H protein 48 h after *Ah* infection. *R. dybowskii*’s Fer-H protein can participate in the immune response after pathogen infection by regulating its own expression. The study lays a theoretical foundation for further research on the regulatory mechanism of *Fer-H* in amphibians’ immune response.

## Supporting information

S1 Fig Original western blot images. (ZIP)

## References

[1] Laufberger V. Sur la cristallisation de la ferritine. Bull Soc Chim Biol. 1937;19(1575):4582.

[2] Chasteen ND, Harrison PM. Mineralization in ferritin: an efficient means of iron storage. J Struct Biol. 1999;126(3):182–94. doi: 10.1006/jsbi.1999.4118 10441528

[3] Liu Q-N, Xin Z-Z, Liu Y, Wang Z-F, Chen Y-J, Zhang D-Z, et al. A ferritin gene from *Procambarus clarkii,* molecular characterization and in response to heavy metal stress and *lipopolysaccharide* challenge. Fish Shellfish Immunol. 2017;63:297–303. doi: 10.1016/j.fsi.2017.02.025 28232280

[4] Chen S, Wu C, Xie Y, Wu Y, Dai S, Wang X, et al. Molecular cloning, characterization and expression modulation of four ferritins in black carp *Mylopharyngodon piceus* in response to *Aeromonas hydrophila* challenge. Aquaculture Reports. 2020;16:100238. doi: 10.1016/j.aqrep.2019.100238

[5] Arosio P, Carmona F, Gozzelino R, Maccarinelli F, Poli M. The importance of eukaryotic ferritins in iron handling and cytoprotection. Biochem J. 2015;472(1):1–15. doi: 10.1042/BJ20150787 26518749

[6] Ruan Y-H, Kuo C-M, Lo C-F, Lee M-H, Lian J-L, Hsieh S-L. Ferritin administration effectively enhances immunity, physiological responses, and survival of Pacific white shrimp (*Litopenaeus vannamei*) challenged with white spot syndrome virus. Fish Shellfish Immunol. 2010;28(4):542–8. doi: 10.1016/j.fsi.2009.12.013 20045064

[7] Deák M, Horváth GV, Davletova S, Török K, Sass L, Vass I, et al. Plants ectopically expressing the iron-binding protein, ferritin, are tolerant to oxidative damage and pathogens. Nat Biotechnol. 1999;17(2):192–6. doi: 10.1038/6198 10052358

[8] Zhang XL, Bi JH, Zhang XD, Ma YW. New distribution of Rana *dybowskii* from Inner Mongolia. Journal of Inner Mongolia Normal University (Natural Science Edition). 2017;46:715–21. doi: 10.3969/j.issn.1001-8735.2017.05.025

[9] Bradford DF. Mass mortality and extinction in a high-elevation population of Rana muscosa. J Herpetol. 1991;25(2):174–7.

[10] Wu T, Liu T, Liu Y, Zhao X, Liu Y, Liu P, et al. Cloning and tissue expression analysis of the LepROT gene of Rana dybowskii. Sheng Wu Gong Cheng Xue Bao. 2022;38(5):1859–73. doi: 10.13345/j.cjb.210615 35611734

[11] Kotla NK, Dutta P, Parimi S, Das NK. The role of ferritin in health and disease: recent advances and understandings. Metabolites. 2022;12(7):609. doi: 10.3390/metabo12070609 35888733 PMC9320524

[12] Densmore CL, Green DE. Diseases of amphibians. ILAR Journal. 2007;48(3):235–54. doi: 10.1093/ilar.48.3.235 17592186

[13] Guénette SA, Giroux M-C, Vachon P. Pain perception and anaesthesia in research frogs. Exp Anim. 2013;62(2):87–92. doi: 10.1538/expanim.62.87 23615302

[14] Smith BD, Vail KJ, Carroll GL, Taylor MC, Jeffery ND, Vemulapalli TH, et al. Comparison of Etomidate, Benzocaine, and MS222 Anesthesia with and without Subsequent Flunixin Meglumine Analgesia in African Clawed Frogs (*Xenopus laevis*). J Am Assoc Lab Anim Sci. 2018;57(2):202–9. 29555009 PMC5868386

[15] Cakir Y, Strauch SM. Tricaine (MS-222) is a safe anesthetic compound compared to benzocaine and pentobarbital to induce anesthesia in leopard frogs (*Rana pipiens*). Pharmacol Rep. 2005;57(4):467–74. 16129913

[16] Hood MI, Skaar EP. Nutritional immunity: transition metals at the pathogen-host interface. Nat Rev Microbiol. 2012;10(8):525–37. doi: 10.1038/nrmicro2836 22796883 PMC3875331

[17] Drakesmith H, Prentice AM. Hepcidin and the iron-infection axis. Science. 2012;338(6108):768–72. doi: 10.1126/science.1224577 23139325

[18] Parrow NL, Fleming RE, Minnick MF. Sequestration and scavenging of iron in infection. Infect Immun. 2013;81(10):3503–14. doi: 10.1128/IAI.00602-13 23836822 PMC3811770

[19] Murdoch CC, Skaar EP. Nutritional immunity: the battle for nutrient metals at the host-pathogen interface. Nat Rev Microbiol. 2022;20(11):657–70. doi: 10.1038/s41579-022-00745-6 35641670 PMC9153222

[20] Lee J-H, Pooley NJ, Mohd-Adnan A, Martin SAM. Cloning and characterisation of multiple ferritin isoforms in the Atlantic salmon (Salmo salar). PLoS One. 2014;9(7):e103729. doi: 10.1371/journal.pone.0103729 25078784 PMC4117605

[21] Wang W, Zhang M, Sun L. Ferritin M of *Cynoglossus semilaevis*: an iron-binding protein and a broad-spectrum antimicrobial that depends on the integrity of the ferroxidase center and nucleation center for biological activity. Fish Shellfish Immunol. 2011;31(2):269–74. doi: 10.1016/j.fsi.2011.05.012 21651984

[22] Elvitigala DAS, Premachandra HKA, Whang I, Oh M-J, Jung S-J, Park C-J, et al. A teleostean counterpart of ferritin M subunit from rock bream (*Oplegnathus fasciatus*): an active constituent in iron chelation and DNA protection against oxidative damage, with a modulated expression upon pathogen stress. Fish Shellfish Immunol. 2013;35(5):1455–65. doi: 10.1016/j.fsi.2013.08.012 23978565

[23] Oh M, Umasuthan N, Elvitigala DAS, Wan Q, Jo E, Ko J, et al. First comparative characterization of three distinct ferritin subunits from a teleost: Evidence for immune-responsive mRNA expression and iron depriving activity of seahorse (*Hippocampus abdominalis*) ferritins. Fish Shellfish Immunol. 2016;49:450–60. doi: 10.1016/j.fsi.2015.12.039 26747640

[24] Ford GC, Harrison PM, Rice DW, Smith JM, Treffry A, White JL, et al. Ferritin: design and formation of an iron-storage molecule. Philos Trans R Soc Lond B Biol Sci. 1984;304(1121):551–65. doi: 10.1098/rstb.1984.0046 6142491

[25] Arosio P, Ingrassia R, Cavadini P. Ferritins: a family of molecules for iron storage, antioxidation and more. Biochim Biophys Acta. 2009;1790(7):589–99. doi: 10.1016/j.bbagen.2008.09.004 18929623

[26] Torti FM, Torti SV. Regulation of ferritin genes and protein. Blood. 2002;99(10):3505–16. doi: 10.1182/blood.v99.10.3505 11986201

[27] Boyd D, Vecoli C, Belcher DM, Jain SK, Drysdale JW. Structural and functional relationships of human ferritin H and L chains deduced from cDNA clones. J Biol Chem. 1985;260(21):11755–61. doi: 10.1016/s0021-9258(17)39094-4 3840162

[28] Arosio P, Yokota M, Drysdale JW. Structural and immunological relationships of isoferritins in normal and malignant cells. Cancer Res. 1976;36(5):1735–9. 57825

[29] Levi S, Yewdall SJ, Harrison PM, Santambrogio P, Cozzi A, Rovida E, et al. Evidence of H- and L-chains have co-operative roles in the iron-uptake mechanism of human ferritin. Biochem J. 1992;288 ( Pt 2)(Pt 2):591–6. doi: 10.1042/bj2880591 1463463 PMC1132051

[30] Guo J, Lyu S, Qi Y, Chen X, Yang L, Zhao C, et al. Molecular evolution and gene expression of ferritin family involved in immune defense of lampreys. Dev Comp Immunol. 2023;146:104729. doi: 10.1016/j.dci.2023.104729 37187445

[31] Chen MD, Zhu K, Xu ZJ, Li CH, Miao L, Chen J. Oceanologia et Limnologia Sinica. 2017;48(2):373–82. doi: 10.11693/hyhz20161000232

[32] Baudrit P, Petitjean A, Potier P, Trosseille X, Vallencien G. Comparison of the Thorax Dynamic Responses of Small Female and Midsize Male Post Mortem Human Subjects in Side and Forward Oblique Impact Tests. Stapp Car Crash J. 2014;58:103–21. doi: 10.4271/2014-22-0004 26192951

[33] Gong J, Sun Q-P, Xue F, Fang S-G, Wan Q-H. Molecular characterization of the major histocompatibility complex class Ia gene in the black-spotted frog, Pelophylax nigromaculata. Biochem Genet. 2013;51(11–12):876–88. doi: 10.1007/s10528-013-9614-9 23835916

[34] Yin GP, Wang LS. PRRSV-GP5 gene cloning and analysis of B cell antigen epitope. J Sichuan Agric Univ. 2016;34(2):234–8. doi: 10.16036/j.issn.1000-2650.2016.02.019

[35] Ling X-D, Dong W-T, Zhang Y, Qian X, Zhang W-D, He W-H, et al. Comparative transcriptomics and histopathological analysis of crucian carp infection by atypical Aeromonas salmonicida. Fish Shellfish Immunol. 2019;94:294–307. doi: 10.1016/j.fsi.2019.09.006 31491530

[36] Salla RF, Jones-Costa M, Abdalla FC, Vidal FAP, Boeing GANS, Oliveira CR, et al. Differential liver histopathological responses to amphibian chytrid infection. Dis Aquat Organ. 2020;142:177–87. doi: 10.3354/dao03541 33331285

[37] Liu D, Geng Y, Wang K, Yu Z, Chen D, Ouyang P, et al. Dynamic pathological lesions and tissue distribution of Chinese giant salamanders infected with CGSRV. Journal of Fishery Sciences of China. 2017;24(1):146. doi: 10.3724/sp.j.1118.2017.16119

[38] Liu T, Guo J, Chen Z, Liu Y, Jing L, Liu P, et al. Identification of heat shock protein hsp70 family genes from Rana amurensis and its expression profiles upon infection. Sheng Wu Gong Cheng Xue Bao. 2023;39(4):1710–30. doi: 10.13345/j.cjb.220641 37154334

[39] Bin-Kamran A, Mishra A, Reddy S, Reddy N, Khan R, Kruger AK. Role of Hepatic Macrophages in Acute and Chronic Injury and Repair. Georgetown Medical Review. 2022;6(1). doi: 10.52504/001c.34718

[40] Zeng JF, Wang J. Pathological observation on Aeromonas hydrophila septicemia of Trionyx sinensis. Natural Science of Hainan University. 2000;18(3):270–2. doi: 10.15886/j.cnki.hdxbzkb.2000.03.013

[41] Shabana SM, Rashed MA, Atia AA, El-Hady HAMA. Characterization of Aeromonas hydrophila isolated from freshwater fish with control trial. Sci Rep. 2025;15(1):32668. doi: 10.1038/s41598-025-19907-6 40987797 PMC12457639

[42] Held KD, Sylvester FC, Hopcia KL, Biaglow JE. Role of Fenton Chemistry in Thiol-Induced Toxicity and Apoptosis. Radiation Research. 1996;145(5):542. doi: 10.2307/3579272 8619019

[43] Luo J, Mills K, le Cessie S, Noordam R, van Heemst D. Ageing, age-related diseases and oxidative stress: What to do next?. Ageing Res Rev. 2020;57:100982. doi: 10.1016/j.arr.2019.100982 31733333

[44] Bresgen N, Eckl PM. Oxidative stress and the homeodynamics of iron metabolism. Antioxid Redox Signal. 2015;23(16):1389–409. doi: 10.1089/ars.2014.611126054376

[45] Hu N, Yu C, Jin J, Zhao X, Zhao Y, Wei H, et al. Impact of photoperiods on the specific activities of immune and antioxidant enzymes in different tissues of Dybowski’s frog (Rana dybowskii). Biological Rhythm Research. 2022;53(11):1790–9. doi: 10.1080/09291016.2022.2043588

[46] Prokić MD, Borković-Mitić SS, Krizmanić II, Mutić JJ, Vukojević V, Nasia M, et al. Antioxidative responses of the tissues of two wild populations of Pelophylax kl. esculentus frogs to heavy metal pollution. Ecotoxicol Environ Saf. 2016;128:21–9. doi: 10.1016/j.ecoenv.2016.02.005 26874985

[47] Mohammadi G, Karimi AA, Hafezieh M, Dawood MAO, Abo-Al-Ela HG. Pistachio hull polysaccharide protects Nile tilapia against LPS-induced excessive inflammatory responses and oxidative stress, possibly via TLR2 and Nrf2 signaling pathways. Fish Shellfish Immunol. 2022;121:276–84. doi: 10.1016/j.fsi.2021.12.042 34968712

[48] Zhang C, Yuan X, Xu R, Qi Q, Wang Y. The intestinal histopathology, innate immune response and antioxidant capacity of blunt snout bream (Megalobrama amblycephala) in response to Aeromonas hydrophila. Fish Shellfish Immunol. 2022;124:525–33. doi: 10.1016/j.fsi.2022.04.037 35489592

[49] Deepika MS, Thangam R, Vijayakumar TS, Sasirekha R, Vimala RTV, Sivasubramanian S, et al. Antibacterial synergy between rutin and florfenicol enhances therapeutic spectrum against drug resistant Aeromonas hydrophila. Microb Pathog. 2019;135:103612. doi: 10.1016/j.micpath.2019.103612 31252064

[50] Kong X, Qiao D, Zhao X, Wang L, Zhang J, Liu D, et al. The molecular characterizations of Cu/ZnSOD and MnSOD and its responses of mRNA expression and enzyme activity to Aeromonas hydrophila or lipopolysaccharide challenge in Qihe crucian carp Carassius auratus. Fish Shellfish Immunol. 2017;67:429–40. doi: 10.1016/j.fsi.2017.06.031 28606861

[51] Sellegounder D, Gupta YR, Murugananthkumar R, Senthilkumaran B. Enterotoxic effects of Aeromonas hydrophila infection in the catfish, Clarias gariepinus: Biochemical, histological and proteome analyses. Vet Immunol Immunopathol. 2018;204:1–10. doi: 10.1016/j.vetimm.2018.08.008 30596375

[52] Boyd JH, Randall SM, Brown AP, Maller M, Botes D, Gillies M, et al. Population Data Centre Profiles: Centre for Data Linkage. Int J Popul Data Sci. 2020;4(2):1139. doi: 10.23889/ijpds.v4i2.1139 32935041 PMC7473267

[53] Boardman GD, Starbuck SM, Hudgins DB, Li X, Kuhn DD. Toxicity of ammonia to three marine fish and three marine invertebrates. Environ Toxicol. 2004;19(2):134–42. doi: 10.1002/tox.20006 15038000

[54] Burýsková B, Hilscherová K, Bláha L, Marsálek B, Holoubek I. Toxicity and modulations of biomarkers in Xenopus laevis embryos exposed to polycyclic aromatic hydrocarbons and their N-heterocyclic derivatives. Environ Toxicol. 2006;21(6):590–8. doi: 10.1002/tox.20222 17091503

[55] Wang F, Fu CX, Chen JS, Chen QH, Liu SL. Biological functions of catalase and its application in animals. Feed Research. 2021;44(5):126–9.

[56] Andrades M, Ritter C, de Oliveira MR, Streck EL, Fonseca Moreira JC, Dal-Pizzol F. Antioxidant treatment reverses organ failure in rat model of sepsis: role of antioxidant enzymes imbalance, neutrophil infiltration, and oxidative stress. J Surg Res. 2011;167(2):e307-13. doi: 10.1016/j.jss.2009.08.005 19959187

[57] Xi L, Wang C, Chen P, Yang Q, Hu R, Zhang H, et al. Expressions of IL-6, TNF-α and NF-κB in the skin of Chinese brown frog (Rana dybowskii). Eur J Histochem. 2017;61(4):2834. doi: 10.4081/ejh.2017.2834 29313598 PMC5656806

[58] Bai L, Li D, Li J, Luo Z, Yu S, Cao S, et al. Bioactive molecules derived from umbilical cord mesenchymal stem cells. Acta Histochem. 2016;118(8):761–9. doi: 10.1016/j.acthis.2016.09.006 27692875

[59] Garcia Neto PG, Titon SCM, Assis VR, Muxel SM, Titon B Jr, Ferreira LF, et al. Immune and endocrine responses of Cururu toads (Rhinella icterica) in their natural habitat after LPS stimulation. Comp Biochem Physiol A Mol Integr Physiol. 2022;269:111213. doi: 10.1016/j.cbpa.2022.111213 35421537

[60] Titon SCM, Titon B Jr, Muxel SM, de Figueiredo AC, Floreste FR, Lima AS, et al. Day vs. night variation in the LPS effects on toad’s immunity and endocrine mediators. Comp Biochem Physiol A Mol Integr Physiol. 2022;267:111184. doi: 10.1016/j.cbpa.2022.111184 35259499

[61] Levi M, Thachil J, Iba T, Levy JH. Coagulation abnormalities and thrombosis in patients with COVID-19. Lancet Haematol. 2020;7(6):e438–40. doi: 10.1016/S2352-3026(20)30145-9 32407672 PMC7213964

[62] Hu Y, Zheng W, Sun L. Identification and molecular analysis of a ferritin subunit from red drum (Sciaenops ocellatus). Fish Shellfish Immunol. 2010;28(4):678–86. doi: 10.1016/j.fsi.2010.01.001 20064620

[63] Sun S, Zhu J, Ge X, Zhang W. Molecular characterization and gene expression of ferritin in blunt snout bream (Megalobrama amblycephala). Fish Shellfish Immunol. 2016;57:87–95. doi: 10.1016/j.fsi.2016.08.029 27539708

[64] Recalcati S, Invernizzi P, Arosio P, Cairo G. New functions for an iron storage protein: the role of ferritin in immunity and autoimmunity. J Autoimmun. 2008;30(1–2):84–9. doi: 10.1016/j.jaut.2007.11.003 18191543

[65] Gozzelino R, Soares MP. Coupling heme and iron metabolism via ferritin H chain. Antioxid Redox Signal. 2014;20(11):1754–69. doi: 10.1089/ars.2013.5666 24124891 PMC3961798

[66] Buracco S, Peracino B, Andreini C, Bracco E, Bozzaro S. Differential effects of iron, zinc, and copper on dictyostelium discoideum cell growth and resistance to *legionella pneumophila*. Front Cell Infect Microbiol. 2018;7:536. doi: 10.3389/fcimb.2017.00536 29379774 PMC5770829

[67] Terán G, Li H, Catrina S-B, Liu R, Brighenti S, Zheng X, et al. High glucose and carbonyl stress impair HIF-1-regulated responses and the control of mycobacterium tuberculosis in macrophages. mBio. 2022;13(5):e0108622. doi: 10.1128/mbio.01086-22 36121152 PMC9600926

[68] Erinmez M, Zer Y. In vitro effects of deferoxamine on antibiotic susceptibility in Gram-negative bacteria. Adv Clin Exp Med. 2024;33(5):491–7. doi: 10.17219/acem/169794 37593774

[69] Cheon Y-I, Kim JM, Shin S-C, Kim H-S, Lee J-C, Park GC, et al. Effect of deferoxamine and ferrostatin-1 on salivary gland dysfunction in ovariectomized rats. Aging (Albany NY). 2023;15(7):2418–32. doi: 10.18632/aging.204641 37036468 PMC10120905

